# Finite element and *in vitro* biomechanical analysis of a novel magnesium degradation-induced variable fixation plate

**DOI:** 10.3389/fbioe.2026.1774985

**Published:** 2026-03-02

**Authors:** Jian Wen, Xingyu Wang, Zhe Wang, Yu Zeng, Xiaofan Chen, Xueqi Liu, Xieping Dong

**Affiliations:** 1 Department of Pain Management, The 2nd Affiliated Hospital, Jiangxi Medical College, Nanchang University, Nanchang, Jiangxi, China; 2 Department of Orthopedics, Jiangxi Province Hospital of Integrated Chinese and Western Medicine, Nanchang, Jiangxi, China; 3 JXHC Key Laboratory of Digital Orthopedics, Jiangxi Provincial People’s Hospital, The First Affiliated Hospital of Nanchang Medical College, Nanchang, Jiangxi, China; 4 Department of Orthopedics, People’s Hospital of Ningxia Hui Autonomous Region, Third Clinical Medical College of Ningxia Medical University, Ningxia Medical University, Yinchuan, China

**Keywords:** biomechanical, finite element analysis, magnesium, micromotion, variable fixation plate

## Abstract

**Background:**

Magnesium degradation-induced variable fixation plates (MVFPs) offer different fixation modes during fracture healing, but their biomechanical reliability is not well established.

**Materials and Methods:**

CT images of femurs from volunteers were used to build a model, and Abaqus software simulated deformation, stress, and relative displacement under various stress conditions. Mechanical tests including vertical loading, four-point bending, torsion, and fatigue were conducted using femur simulation models and suitable magnesium shims were screened.

**Results:**

Finite element analysis showed that under 700N vertical loading, MVFP exhibited 83%–116% of the total deformation, 88%–120% of the maximum stress, and 86%–121% of the average relative displacement compared to locking plate (LP). Under 250N four-point bending, these were 76%–186%, 73%–183%, and 61%–170%, respectively. Under 10Nm torsional moment, they were 102%–109%, 114%–118% (for implants), and 110%–113%, respectively. *In vitro* biomechanical tests showed that MVFP had greater total and relative displacements but lower axial, four-point bending, and torsional stiffness (81.5%, 68.5%, and 63.9% of LP, respectively). Fatigue testing indicated both LP and MVFP samples endured 100,000 cycles of 700N vertical load without failure. The MVFP with a 0.5 mm shim exhibited superior stiffness and offered greater space for elastic deformation compared to the 1 mm shim.

**Conclusion:**

Although MVFP’s stiffness slightly decreases compared to LP after shim degradation, it improves interfragmentary micromotion and reduces stress shielding while maintaining good fatigue resistance. MVFP with 0.5 mm axial micromotion shows promise for further development and clinical application.

## 1 Introduction

A large number of studies have shown that axial micromotion between fracture ends with a low frequency of 0.2–1 mm is beneficial to fracture healing (Hofmann-Fliri et al., 2020; Han et al., 2020; Plecko et al., 2020; Bottlang et al., 2016; Elkins et al., 2016; Gardner et al., 2010; Goodship and Kenwright, 1985; Claes et al., 1995; Claes et al., 1997; Bottlang et al., 2010). Currently, devices designed to facilitate micromotion at the fracture site, such as axial micromovement plates, distal cortical screws, and biphasic plates, have shown promising results in preclinical trials (Lv et al., 2025; Wen et al., 2024; Wen et al., 2026). However, recent research indicates that the initial mechanical stability of fracture ends, particularly within the first 4–8 days, is crucial for effective vascularization and bone regeneration (Liu et al., 2018; Gardner et al., 2006; Lienau et al., 2005; Claes et al., 2002; Wallace et al., 1994). Therefore, we designed a magnesium degradation-induced variable fixation plate (MVFP). It provides strong initial fixation to support early-stage healing and gradually transitions to axial micromotion fixation as the magnesium shim degrades, thereby offering adaptable fixation modes at different stages of fracture healing.

In this study, the femoral MVFP was designed based on the locking plate (LP), sharing similar appearance, size, and weight. However, the MVFP comprises three separate components: the plate subject, a slider, and a magnesium shim, whereas the LP is a single integrated unit. This design difference impacts stress distribution and force transfer. Therefore, further investigation is needed to determine whether the MVFP can meet the biomechanical requirements for effective internal fixation of fractures.

Finite Element Analysis (FEA) is an engineering method that breaks down complex structures into many small, simple geometric units, each with defined geometric and physical properties. These units are combined using the Finite Element method to approximate the behavior of the entire structure, allowing for simulation and evaluation under various conditions (Knowles, 1984). In the medical field, FEA enhances understanding of biological systems’ mechanical behavior, improves the safety and efficiency of medical devices and treatments, and supports research and clinical practice (Salaha et al., 2023; Rieger et al., 1990). This study aims to assess the biomechanical properties and safety of MVFP fixation for femoral fractures using FEA. Additionally, *in vitro* biomechanical experiments were conducted which are performed in a controlled environment and can provide valuable data on biological mechanics. This study implemented staged adaptive fixation by integrating degradable magnesium sheets, a strategy not commonly explored in conventional steel locking plates. Through a combined finite element and experimental biomechanics approach under multiple loading conditions, we evaluated the MVFP’s biomechanical behavior, efficacy, and safety across various stress environments (Yenna et al., 2011; Scolaro et al., 2014; Crump et al., 2020). Consequently, this work provides essential data supporting the clinical translation of the MVFP for accelerated and improved fracture healing.

## 2 Materials and methods

### 2.1 FEA

#### 2.1.1 Modeling

Three healthy adult male volunteers of Han Chinese ethnicity (weight approximately 60–80 kg, height 170–180 cm, BMI between 18.5 and 28 kg/m^2^) were enrolled. They had no significant underlying diseases and were screened to exclude any history of femoral fractures, osteoporosis, bone tumors, tuberculosis, or metabolic disorders. CT data of the left femur were collected and used to perform three-dimensional reconstruction and surface modeling using Materialise Mimics Research 19.0 and Geomagic Studio 2013, respectively. A 10 mm bone segment was excised from the mid-femoral shaft to create a bone defect model, eliminating any effects from bone support on the experimental results. Parameters for the femoral LP and screws produced by Suzhou Kangli Orthopaedic Instrument Co., Ltd. Were obtained and used to create a three-dimensional model with SolidWorks 2019. Fine details of the bone plate and screws, such as locking holes and threads, were simplified. The MVFP with no shim (MVFP^0^), 0.5 mm (MVFP^0.5^), and 1 mm (MVFP^1^) magnesium shim were modeled using the same method. The bone plate and screws were then assembled on the femur through translation and Boolean operations.

#### 2.1.2 Meshing

The assembly was imported into abaqus 6.12 software (Dassault Systèmes, France), the mesh type was tetrahedral, the cell type was C3D10, the global mesh size was 2 mm on the femur and 1 mm on the bone plates and screws, and the volume meshing was performed using the software’s built-in meshing function.

#### 2.1.3 Attribute assignment

According to the literature report (Kovács et al., 2023; Ding et al., 2023; Ding et al., 2022) and the material properties on the MatWeb website (https://matweb.com/), each part is assigned the corresponding material properties (Table 1).

**TABLE 1:** Properties of each material used in the finite element data.

Materials	Density	Young’s modulus	Poisson’s ratio
Titanium alloy (TC4)	4,429 kg/m^3^	111.2 GPa	0.3387
Cortical bone	1,850 kg/m^3^	16.7 GPa	0.3
Cancellous bone	1,500 kg/m^3^	0.028 GPa	0.33
Magnesium	1,800 kg/m^3^	45 GPa	0.35

#### 2.1.4 Boundary conditions

**FIGURE 1:**
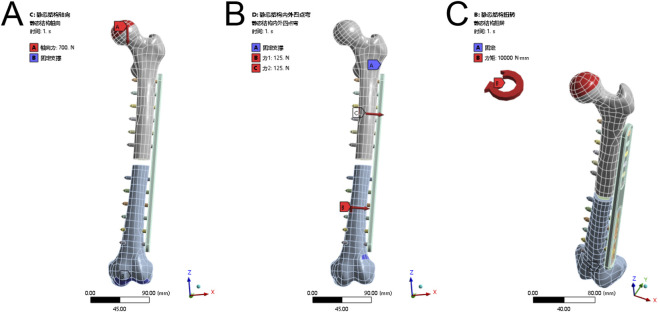
Load and boundary conditions in FEA. **(A)** Vertical loading experiment; **(B)** Internal and external four-point bending test; **(C)** Torsion experiments.

This study simulates loads and constraints for a 70 kg adult, including a single-foot load (Hu et al., 2022; Li et al., 2023; He et al., 2022), 250 N eversion stress (four-point bending) (Sundar et al., 1989; Lenz et al., 2016; Park et al., 2011), and 10 N·m torsional torque (Han et al., 2020; He et al., 2022; Takamura et al., 2022). Figure 1 illustrates the loading and constraint methods (using ANSYS interface; calculations are performed with Abaqus). Contacts are set as follows: binding between cancellous and cortical bone, plate/slider and screw, and screw with both types of bone; friction with a coefficient of 0.1 between the slider and the bone plate, and between the magnesium shim and both the slider and the bone plate.

#### 2.1.5 Solution and post-processing

**FIGURE 2:**
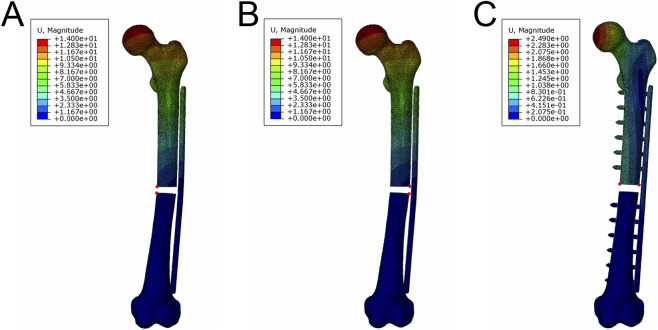
Location of marked points at the broken ends of the fracture (red points). Location of marked points at the plate side **(A)** and non-plate side **(B)** in the vertical loading and four-point bending experiments. **(C)** Location of marked points in the torsion test.

Stress and deformation analysis were used to predict the risk of internal implant failure. Relative displacements at the fracture ends were measured before and after loading to determine if the movement amplitude was suitable for healing (Figures 2A–C).

### 2.2 *In vitro* biomechanical analysis

Axial compression, four-point bending, and torsion tests were conducted to evaluate the resistance of an artificial femoral fracture model fixed with a plate under three FEA simulation scenarios. A fatigue test assessed whether the plate’s performance met orthopedic implant standards, ensuring it could withstand the functional loads expected for patients 3 months post-surgery (Nakhaei et al., 2023). Considering that the MVFP shim is small and degrades within 7–14 days post-implantation, with the fracture subsequently being stabilized by the shim-free MVFP until healing, the biomechanical properties of the shim-free MVFP and LP were compared in *in vitro* simulated experiments.

Ten femoral models (sawbones, Washington, USA, item number: #3406) were purchased and randomly divided into MVFP and LP groups, with five models in each (Bariteau et al., 2014; Zdero et al., 2023). The midpoint of the femoral shaft (The midpoint of the line between the tip of the greater trochanter of the femur and the lateral condyle of the femur) was marked, and either LP or MVFP^1^ was positioned and secured with two bone holders laterally on the femoral shaft with this mark as the center. A power drill and matching drill bit were used to create the nail path along the locking sleeve of the bone plate. After removing the plate, a 10 mm bone segment in the middle of the femoral shaft was excised using an osteotomy guide. The fracture was reduced and fixed with screws along the prefabricated screw path. Finally, the magnesium shim of the MVFP was removed.

All specimens were subjected to axial compression, four-point bending, torsion and fatigue tests in a dry room temperature environment.

Sample Preparation: A 25 mL denture base resin (Shanghai New Century Dental, Type II, Class I) was used to embed the femoral condyle side of each specimen, aligning the line from the center of the femoral head to the femoral condyle perpendicularly to the container’s bottom plane.

Axial compression experiment: After the markers were attached to the fracture ends, the specimens were tested on a universal material testing machine (UTM5105, Shenzhen S&T Co., Ltd.) with a loading speed of 5 mm/min and a maximum load of 700N. Results were recorded as load-displacement curves, and relative displacement at the fracture points was measured using a visual strain meter (BLUEBOX-S, Shenzhen HSEM Technology Co., Ltd.).

Four-point bending test: Specimens were positioned on a universal testing machine with a 72 mm upper span and a 144 mm lower span, oriented downward. A maximum load of 250N was applied at a rate of 5 mm/min, and the results were recorded as load-displacement curves. The relative displacement at the fracture points was also measured using a visual strain meter.

Torsion test: After being secured in the torsion machine (50T 50N·m, Instvik, Suzhou, China), the specimens were tested at a rotation speed of 10°/min and a maximum torque of 10 N·m. Results were recorded as load-displacement curves.

Fatigue test: The distal femur was fixed on an Instron E3000 fatigue testing machine, ensuring that vertical loading aligned with the femur’s force line. Test parameters were: maximum loading force of 700 N, frequency of 2 Hz, sine waveform, load ratio of 0.1, and 100,000 cycles (Bottlang et al., 2010; Nakhaei et al., 2023). The experiment was terminated under the following conditions: 1) Femur fracture; 2) Contact between broken ends; 3) Significant plastic deformation, cracks, or fractures of internal fixation; 4) Fixation failure due to plate or screw dislocation or pullout; 5) Reaching the maximum cycle count. The gap between the fracture ends on the non-plate side was measured with a vernier caliper before and after testing, and the difference was calculated.

#### 2.2.1 *In vitro* biomechanical experiments to screen magnesium shims

Crescent-shaped magnesium shims with thicknesses of 1 mm, 0.75 mm, 0.5 mm, and 0.25 mm were fabricated based on the gap between the MVFP plate and the slider. Biomechanical properties of the MVFP with these shims and with no shim were tested using axial compression experiment with a preloading force of 50 N, a loading speed of 5 mm/min, and a maximum loading of 700 N. The load-displacement curves and maximum displacement were recorded and analyzed using paired one-way ANOVA (GraphPad Prism 8.3.0).

## 3 Results

### 3.1 The deformation and stress distribution maps of the complex under a 700 N axial compressive force using FEA

**FIGURE 3:**
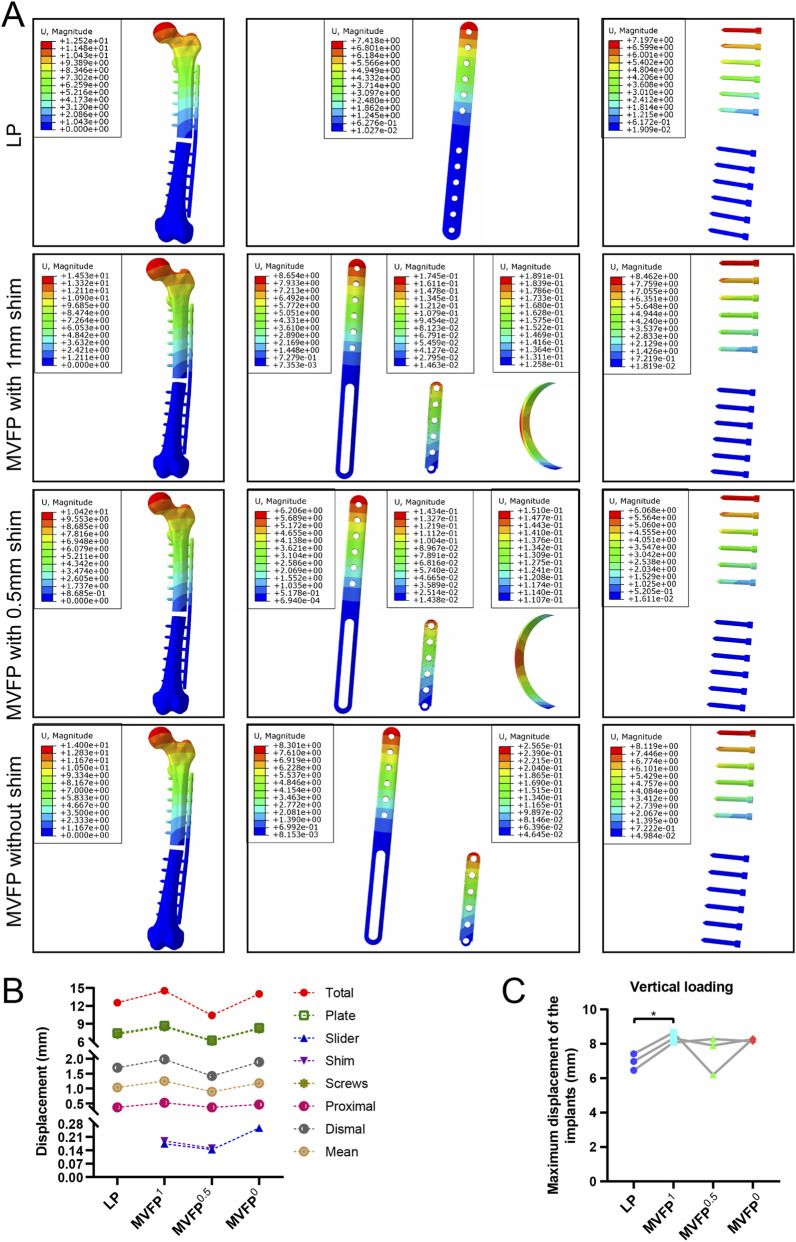
Deformation map, maximum deformation, and relative displacement of markers at fracture ends under 700 N axial pressure: **(A)** Deformation map. **(B)** Dot plot of maximum deformation and relative displacement of the markers. **(C)** Dot plot of maximum deformation by group (one-way ANOVA for paired samples, n = 12); significance levels: unlabeled: no statistical difference; *p < 0.05; **p < 0.01.

Under a 700 N axial compression force, deformation increased towards the femoral head and decreased towards the distal femur (Figure 3A). Among the four fixation methods, MVFP^0.5^ had the smallest total deformation at 10.42 mm, about 83% of that in the LP group (Figure 3B), while MVFP^1^ had the largest at 14.53 mm. Deformation trends for the implants in each group mirrored those of the overall complex. The largest average relative displacement of the fracture ends was 1.25 mm in the MVFP^1^ group, the smallest was 0.89 mm in the MVFP^0.5^ group, and 1.03 mm in the LP group. Calculations from femur fracture models of three volunteers showed MVFP^1^ had significantly greater maximum deformation than LP under 700 N axial pressure (Figure 3C).

**FIGURE 4:**
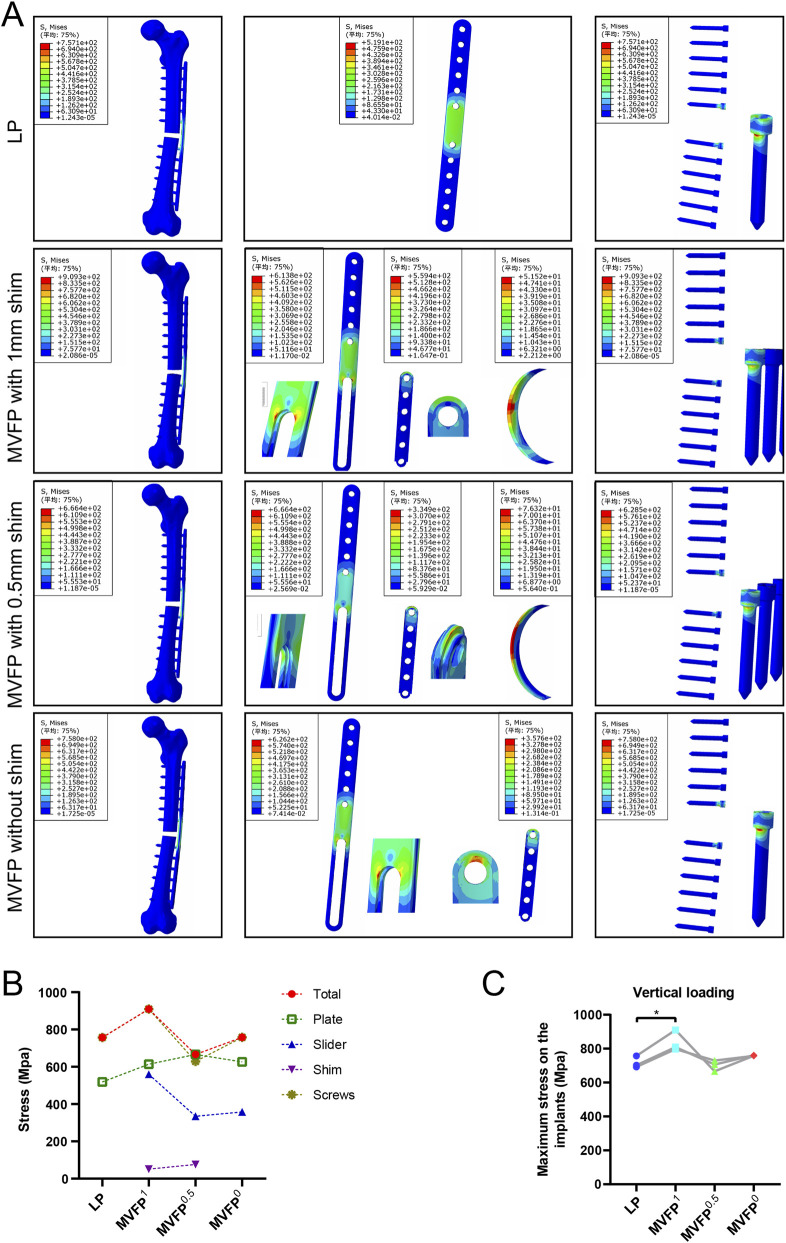
Stress distribution and maximum stresses under 700 N axial pressure: **(A)** Stress distribution map. **(B)** Dot plot of maximum stress. **(C)** Dot plot of maximum stress by group (one-way ANOVA for paired samples, n = 12); significance levels: unlabeled: no statistical difference; *p < 0.05; **p < 0.01.

Under 700 N axial pressure, stress was higher in the middle of the plate and lower on the bone, with relatively uniform distribution across fixation methods (Figure 4A). Maximum stress for LP, MVFP^1^, and MVFP^0^ was at the junction between the screw nut and body, while for MVFP^0.5^, it was at the bone plate bifurcation near the slider. The LP group had the lowest maximum stress at 519.1 MPa, while MVFP^1^, MVFP^0.5^, and MVFP^0^ had stresses 118%, 128%, and 121% higher, respectively (Figure 4B). Calculations from femoral fracture models of three volunteers showed that MVFP^1^ experienced significantly greater maximum stress than LP under 700 N axial pressure (Figure 4C).

### 3.2 The deformation and stress distribution maps of the complex under a 250 N four-point bending load using FEA

**FIGURE 5:**
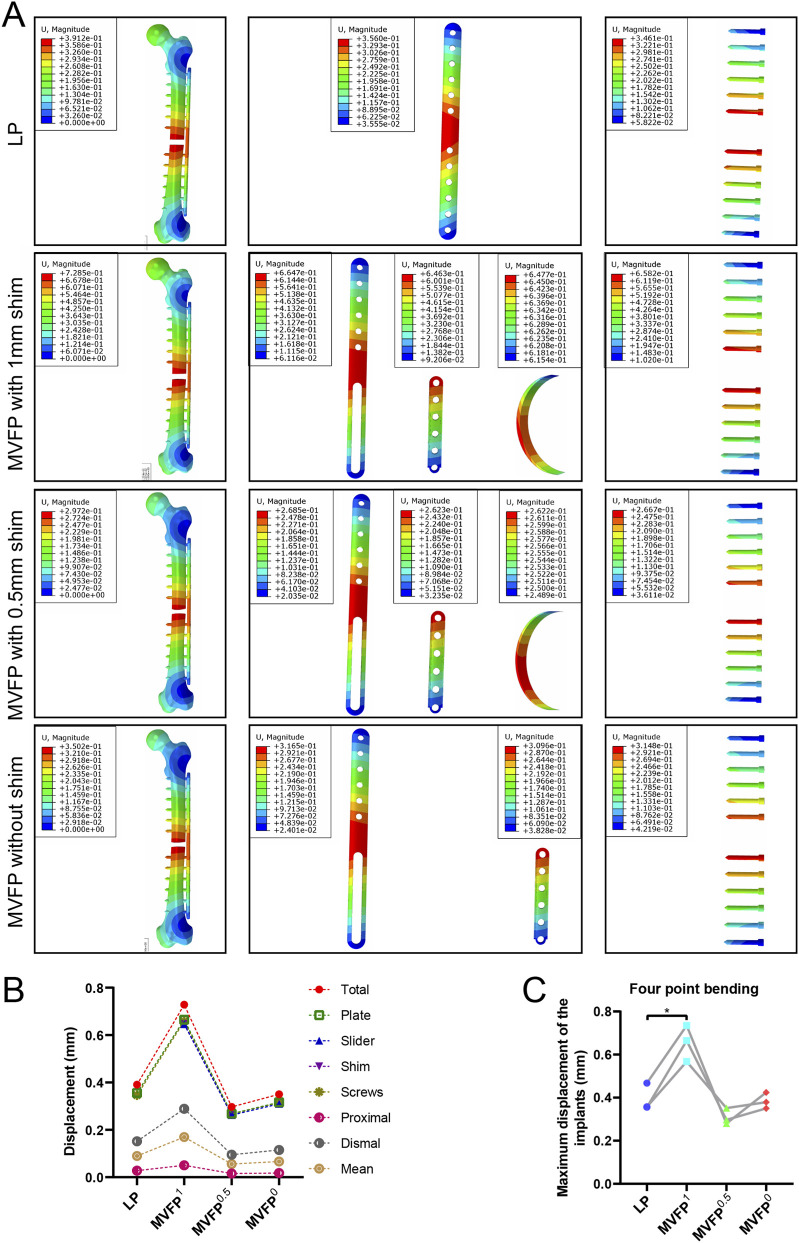
Deformation map, maximum deformation, and relative displacement of markers at fracture ends under 250 N bending stress: **(A)** Deformation map. **(B)** Dot plot of maximum deformation and relative displacement of the markers. **(C)** Dot plot of maximum deformation by group (one-way ANOVA for paired samples, n = 12); significance levels: unlabeled: no statistical difference; *p < 0.05; **p < 0.01.

Under a 250 N four-point bending force, deformation increased with distance from the fixation position, with the least deformation near the fixation (Figure 5A). Among the four fixation methods, MVFP^0.5^ had the smallest total deformation at 0.2972 mm, about 76% of that of the LP group. Conversely, MVFP^1^ showed the highest overall deformation, approximately 186% of the LP group (Figure 5B). Deformation trends for implants mirrored overall complex deformation. The MVFP^1^ group had the greatest average relative displacement at the fracture end, while MVFP^0.5^ had the smallest. Calculations from femoral fracture models of three volunteers revealed that MVFP^1^ experienced significantly greater maximum deformation than LP under 250 N bending stress (Figure 5C).

**FIGURE 6:**
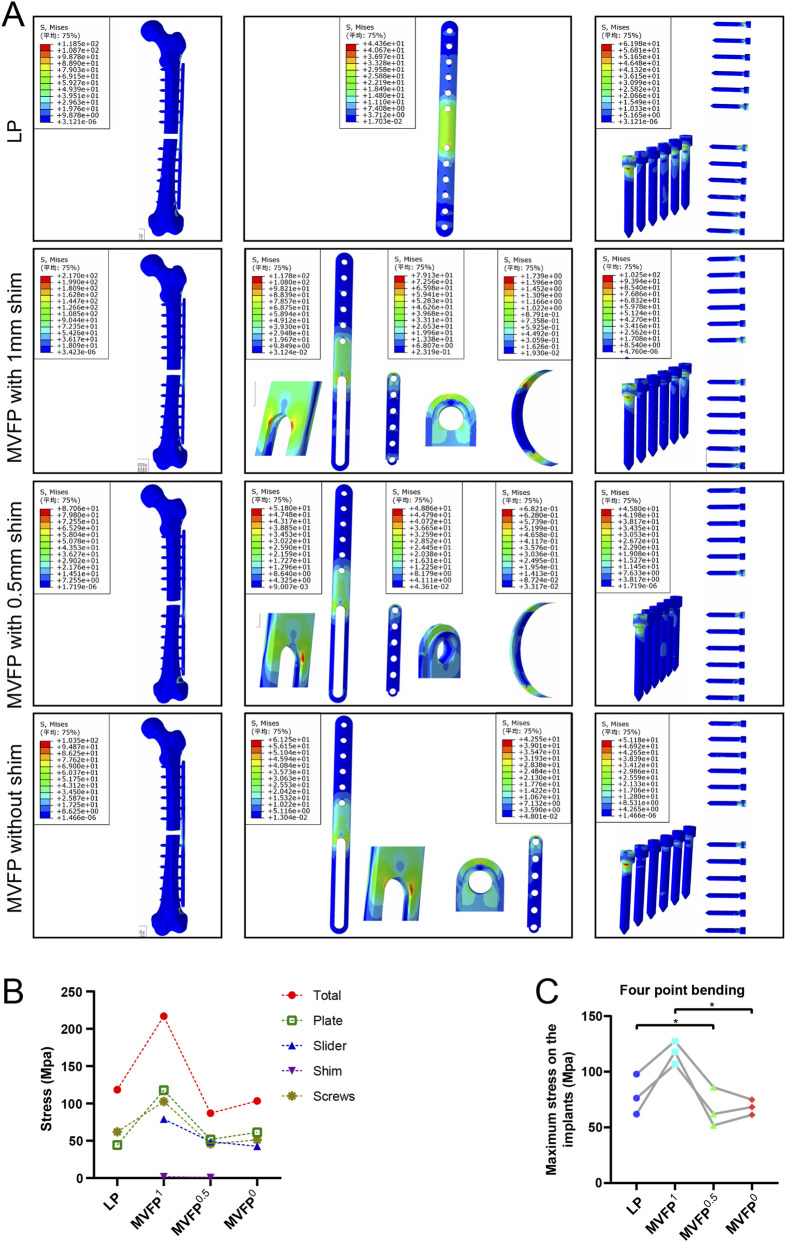
Stress distribution and maximum stresses under 250 N bending stress: **(A)** Stress distribution map. **(B)** Dot plot of maximum stress. **(C)** Dot plot of maximum stress by group (one-way ANOVA for paired samples, n = 12); significance levels: unlabeled: no statistical difference; *p < 0.05; **p < 0.01.

Under 250 N bending stress, stress was highest on the plate and screw and lowest on the bone. In the LP group, stress was concentrated around the two central screw holes, while in the MVFP group, stress focused at the plate’s middle bifurcation and the junction of the nut and screw (Figure 6A). MVFP^1^ had the highest maximum stress at 217 MPa, while MVFP^0.5^ had the lowest (Figure 6B). Implant stress followed the same trend. Calculations from femur models of three volunteers showed MVFP^1^ had significantly higher maximum stress than MVFP^0^, while MVFP^0.5^ had lower stress than the LP group under 250 N bending stress (Figure 6C).

### 3.3 The deformation and stress distribution maps of the complex under a 10 N·m torque in FEA

**FIGURE 7:**
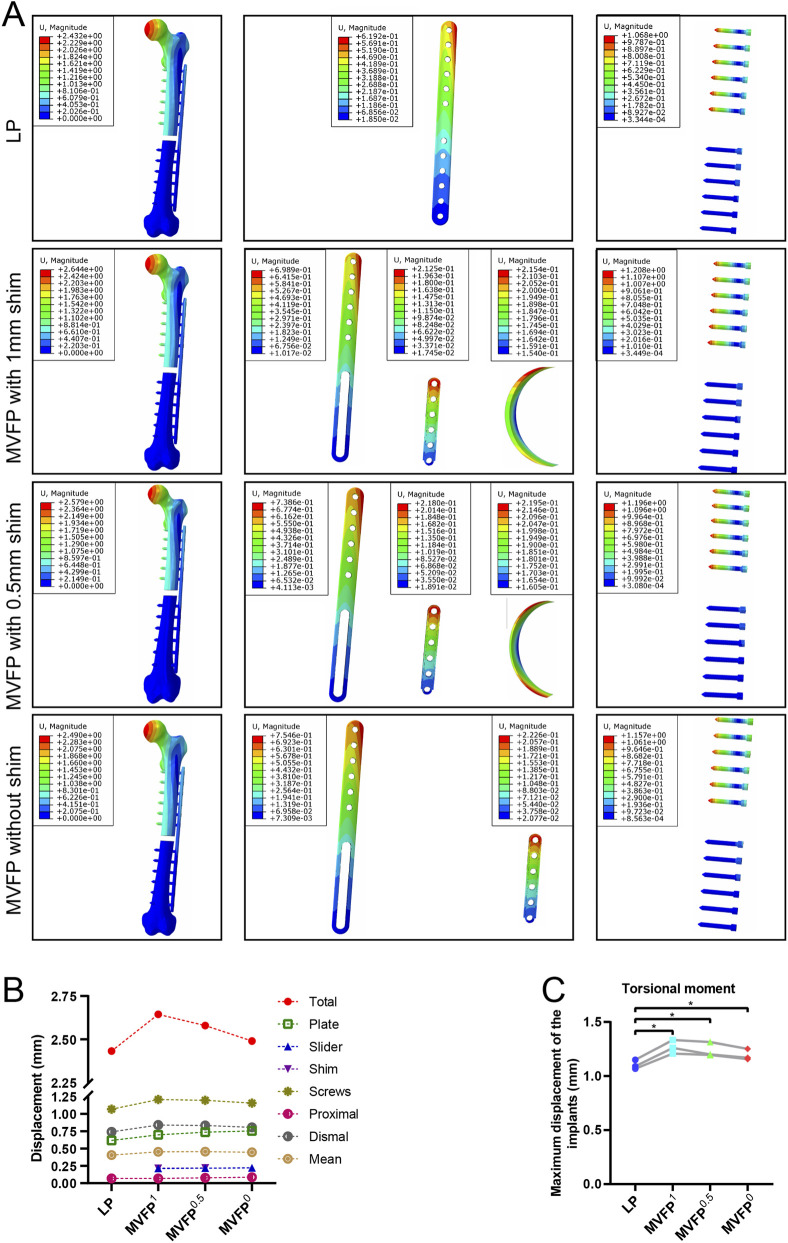
Deformation map, maximum deformation, and relative displacement of markers at fracture ends under 10 N·m torsional stress: **(A)** Deformation map. **(B)** Dot plot of maximum deformation and relative displacement of the markers. **(C)** Dot plot of maximum deformation by group (one-way ANOVA for paired samples, n = 12); significance levels: unlabeled: no statistical difference; *p < 0.05; **p < 0.01.

Under a 10 N·m torque, femur deformation mirrored the longitudinal contour, with the largest deformation at the femoral head. In the MVFP group, the slider’s deformation retained a transverse contour (Figure 7A). Among the four fixation methods, the LP group exhibited the smallest overall deformation at 2.432 mm, while the MVFP^1^ group had the largest deformation, 109% of the LP group’s (Figure 7B). The average marker displacement at the fracture end was 0.406 mm in the LP group and 113% in the MVFP^1^ group. Calculations from femoral fracture models of three volunteers showed that all MVFP groups experienced significantly greater maximum deformation than LP under 10 N·m torsional stress (Figure 7C).

**FIGURE 8:**
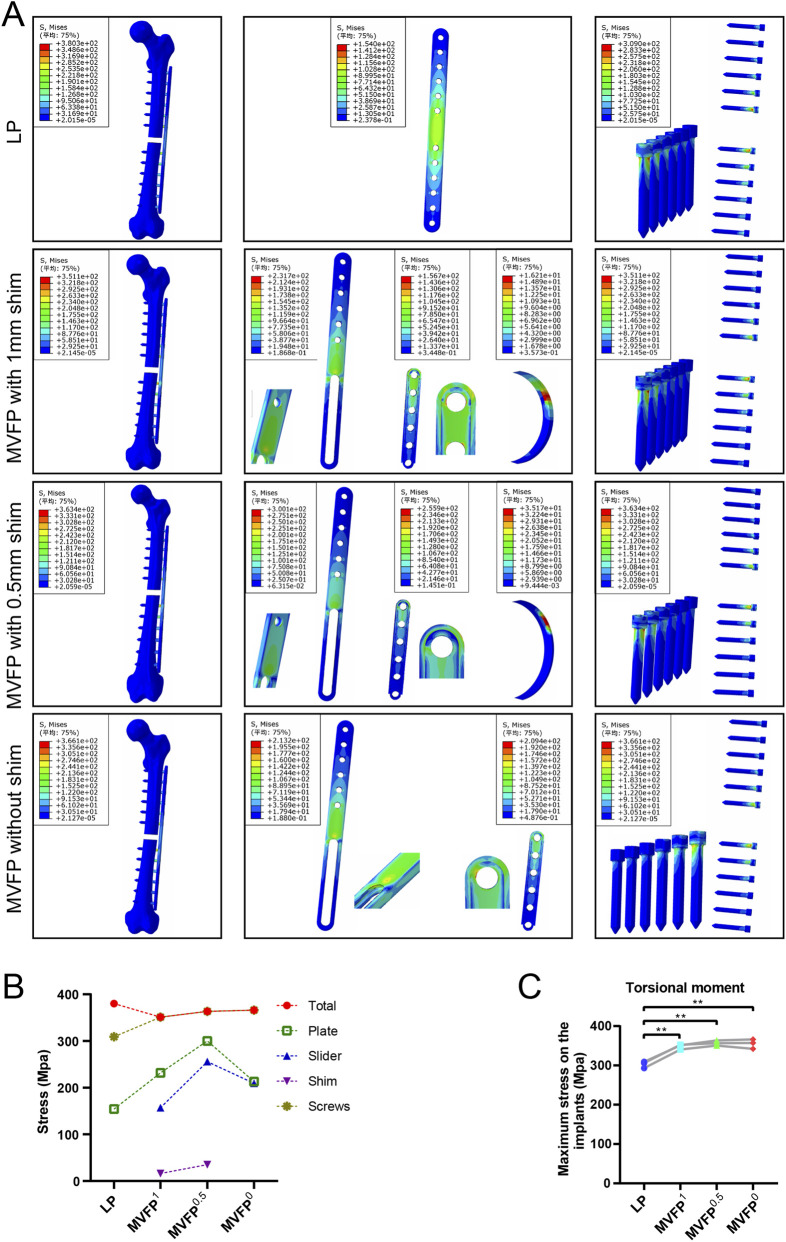
Stress distribution and maximum stresses under 10 N·m torsional stress: **(A)** Stress distribution map. **(B)** Dot plot of maximum stress. **(C)** Dot plot of maximum stress by group (one-way ANOVA for paired samples, n = 12); significance levels: unlabeled: no statistical difference; *p < 0.05; **p < 0.01.

Under 10 N·m torsional stress, stress was highest in the middle of the plate for all groups, with LP showing a more extensive and uniform distribution compared to MVFP (Figure 8A), indicating better torsional resistance for LP. The LP group experienced the highest overall stress and MVFP^1^ the lowest. The maximum stress on the implant was smallest in the LP group and 114%–118% of that in the MVFP group (Figure 8B). In each group, the highest implant stress was at the junction between the screw cap and body. Calculations from femur models of three volunteers revealed that all MVFP groups experienced significantly greater maximum stress than LP under 10 N·m torsional stress (Figure 8C).

### 3.4 Axial compression experiment

**FIGURE 9:**
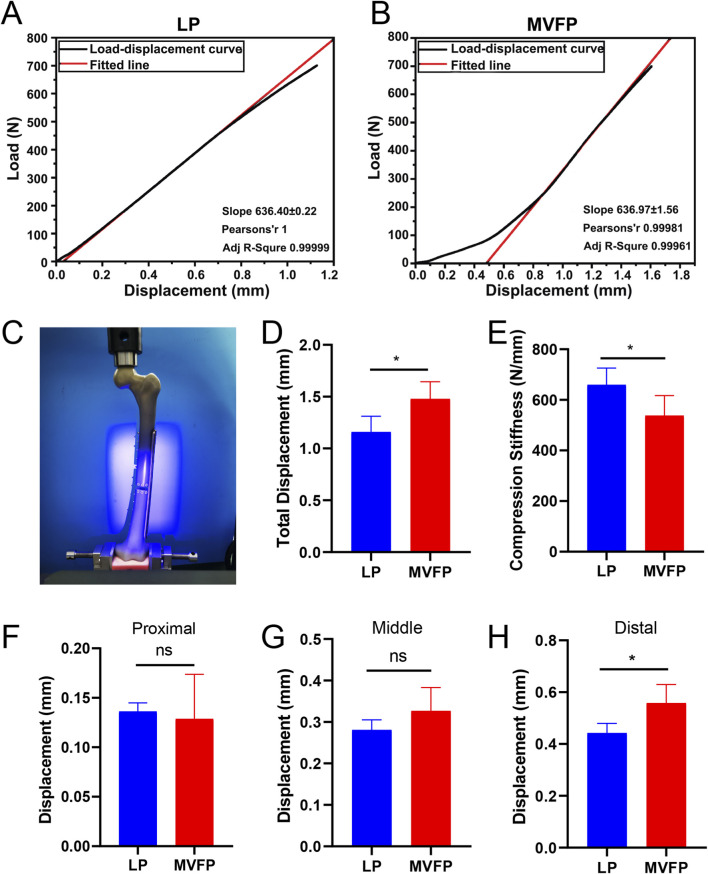
Results of the vertical compression experiment. **(A)** Load-displacement curves for LP group specimens. **(B)** Load-displacement curves for MVFP group specimens. **(C)** Image of a sample post-vertical loading test. Bar plots of the total deformation **(D)**, compression stiffness **(E)** and relative displacements of the fracture ends at the proximal **(F)**, middle **(G)**, and distal **(H)** plate sides. Significance levels: ns (no statistical difference); * (p < 0.05).

Axial compression experiments showed that the average total displacements were 1.16 ± 0.15 mm for the LP group and 1.48 ± 0.16 mm for the MVFP group, with axial loading stiffness of 659.9 ± 65.91 N/mm and 537.5 ± 79.4 N/mm, respectively (81.5% of LP) (Figures 9A–E). In the MVFP group, relative displacements of the fracture ends were 0.13 ± 0.05 mm (proximal), 0.33 ± 0.06 mm (middle), and 0.56 ± 0.07 mm (distal). For the LP group, these displacements were 0.14 ± 0.01 mm (proximal), 0.28 ± 0.02 mm (middle), and 0.44 ± 0.04 mm (distal) (Figures 9F–H). The only significant difference between the groups was in the distal plate side displacement.

### 3.5 Four-point bending test

**FIGURE 10:**
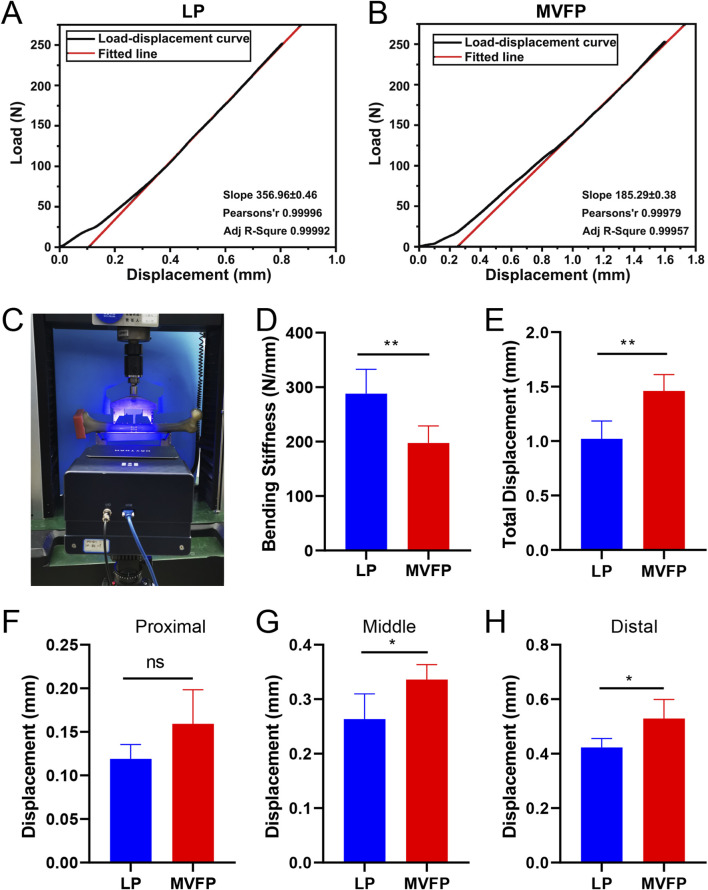
Results of four-point bending experiments. **(A)** Load-displacement curves for LP group specimens. **(B)** Load-displacement curves for MVFP group specimens. **(C)** Image of a sample after the four-point bending test. Bar plots of the total deformation **(D)** bending stiffness **(E)** and relative displacements at the proximal **(F)** middle **(G)** and distal **(H)** plate sides. Significance levels: ns (no statistical difference); * (p < 0.05); ** (p < 0.01).

The four-point bending test results showed average total displacements of 1.02 ± 0.16 mm for the LP group and 1.46 ± 0.15 mm for the MVFP group. The bending stiffness was 288.1 ± 44.58 N/mm for LP and 197.4 ± 31.38 N/mm for MVFP (68.5% of LP) (Figures 10A–C). Differences in total displacement and bending stiffness between the two groups were statistically significant (*P* < 0.01) (Figures 10D,E). In the MVFP group, relative displacements at the fracture end were 0.16 ± 0.04 mm (proximal), 0.34 ± 0.03 mm (middle), and 0.53 ± 0.07 mm (distal). In the LP group, these were 0.12 ± 0.02 mm (proximal), 0.26 ± 0.05 mm (middle), and 0.42 ± 0.03 mm (distal) (Figures 10F–H). Significant differences were found in the middle and distal plate displacements between the two groups (*P* < 0.05).

### 3.6 Torsion and fatigue test

**FIGURE 11:**
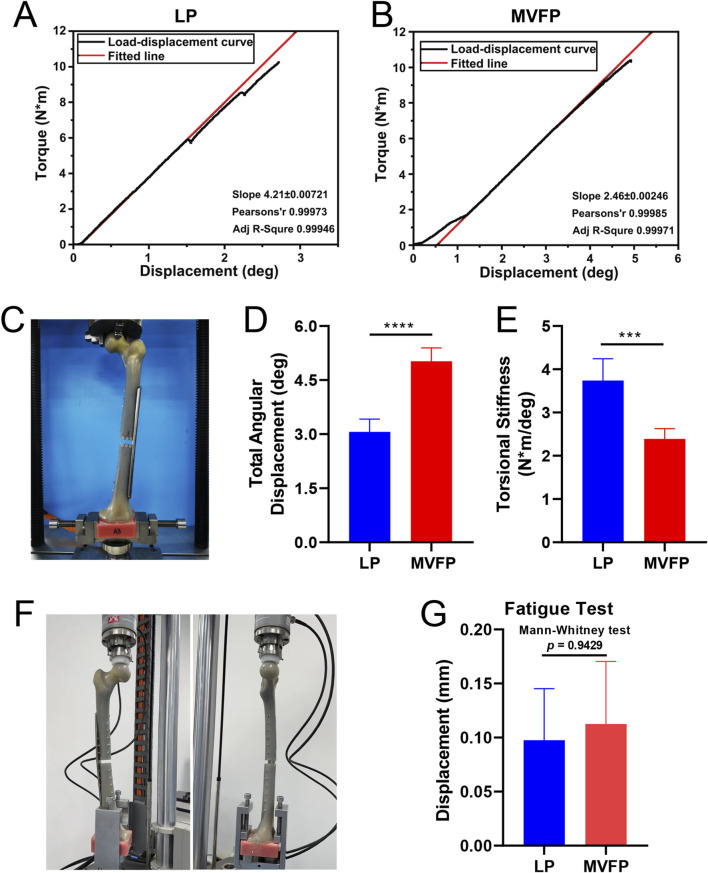
Results of torsion and fatigue tests. **(A)** Load-displacement curves for LP group specimens. **(B)** Load-displacement curves for MVFP group specimens. **(C)** Image of a sample after the torsion test. Bar plots of the total angular displacement **(D)** and torsional stiffness **(E)** for the torsion test. **(F)** Image of a specimen post-fatigue test. **(G)** Bar plot of the average relative displacement of markers after the fatigue test. Significance levels: ns (no statistical difference); * (p < 0.05); ** (p < 0.01); *** (p < 0.001); **** (p < 0.0001).

The torsion test revealed that the MVFP group had a higher average total angular displacement and lower average torsional stiffness compared to the LP group (Figures 11A–E). Specifically, the average total angular displacements were 3.06° ± 0.36° for LP and 5.02° ± 0.37° for MVFP. Torsional stiffness values were 3.74 ± 0.51 Nm/° for LP and 2.39 ± 0.24 Nm/° for MVFP (about 63.9% of LP). The fatigue test results showed that both groups could withstand a cyclic vertical load of 700N (equivalent to body weight) for 100,000 cycles (Figure 11F), with no significant difference in the relative displacement of the markers at the fracture ends between the groups (Figure 11G).

### 3.7 Magnesium shim screening

**FIGURE 12:**
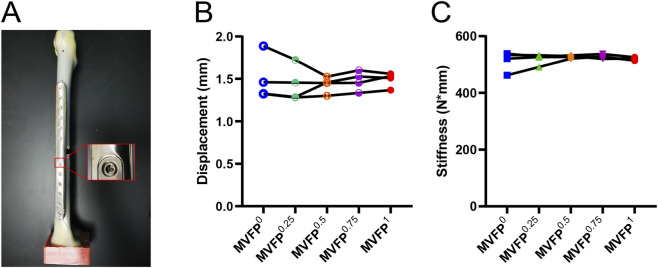
Screening of magnesium shims with varying thicknesses: **(A)** MVFP assembly with a magnesium shim; **(B)** Displacement of MVFP assemblies with shims of different thicknesses under a 700N vertical load; **(C)** Stiffness of MVFP assemblies with shims of varying thicknesses under a 700N vertical load.

The average displacement of MVFP samples with varying magnesium shim thicknesses under a 700N axial load was: 1.492 mm (1 mm), 1.479 mm (0.75 mm), 1.434 mm (0.5 mm, minimum), 1.437 mm (0.25 mm), and 1.499 mm (0 mm, maximum) (Figures 12A,B). Average stiffness values were: 518.9 N/mm (1 mm), 527.7 N/mm (0.75 mm, maximum), 527.4 N/mm (0.5 mm), 519.5 N/mm (0.25 mm), and 513.7 N/mm (0 mm, minimum) (Figure 12C). Statistical analysis found significant differences in displacement (*P* = 0.0019) and stiffness (*P* = 0.0476) among groups. Tukey’s test revealed MVFP^0.5^ samples had significantly smaller displacement and greater stiffness than MVFP^1^ samples (*P* = 0.0436 and *P* = 0.0268, respectively). These findings align with FEA results, which also showed MVFP^0.5^ samples had less displacement than MVFP^0^ and MVFP^1^ samples.

## 4 Discussion

FEA results indicate no significant stress concentrations or intensities exceeding the material’s yield strength in the MVFP. Compared to the LP plate’s more uniform stress distribution due to its monolithic structure, the MVFP plate shows 18%–28% higher maximum stress under a 700N axial load, though it remains below the titanium alloy’s yield strength. Both LP and MVFP exhibit increased stress at the junction between the screw nut and body, a biomechanically weak point. Notably, the maximum stress on the MVFP^0.5^ screw is only 83% of that in the LP group, suggesting better performance. Under a 250N four-point bending load, MVFP^0.5^ shows slightly lower deformation and maximum stress compared to the LP, whereas MVFP^1^ shows higher stress and MVFP^0^ is similar to LP. Thus, a 0.5 mm gap may be optimal. However, LP outperforms MVFP in anti-torsion performance, with MVFP’s maximum stress being 114%–118% of LP’s.

During the gradual weight-bearing postoperative process, the plate’s load is closely related to the patient’s weight, with elastic deformation increasing accordingly. The plate fixation is biased, causing minimal deformation near the plate and greater deformation on the opposite side. In *in vitro* biomechanical tests, a 700N vertical load resulted in a 0.14 mm relative displacement at the plate side in the LP group, lower than the range beneficial for fracture healing. Clinical practice often shows even less deformation due to protective weight-bearing. The MVFP group, with lower axial stiffness, showed slightly higher axial displacement compared to the LP group. However, this elastic deformation is influenced by patient weight and rehabilitation strategy rather than being controllable. Then, variable fixation control has the great advantage of being controllable and providing consistent axial micromotion on both the plate and non-plate sides. Therefore, the interfragmentary micromotion should include the axial elastic deformation of the plate and the axial micromotion caused by the micromotion gap. In this study, MVFP^0.5^ demonstrated less elastic deformation under the same load and provided an additional 0.5 mm deformation space, aligning better with micromotion plate requirements.

In *in vitro* biomechanical experiments, the MVFP specimens exhibited 81.5% of the axial compression, 68.5% of the four-point bending, and 63.9% of the torsional stiffness of the LP specimens. Bottlang M et al. found that micromotion fixation with far cortical locking can reduce plate stiffness by 80%–88% and enhance callus proliferation (Bottlang and Feist, 2011). In this study, the MVFP’s axial stiffness was about 81.5% of that of the LP, similar to the stiffness of far cortical locking. Notably, cortical bone in adults has a Young’s modulus of 10–18 GPa, while titanium alloys used in implants have a modulus of around 110 GPa, making them 6–11 times stiffer (Zhou and Yang, 2022; Hashemi et al., 2021). To avoid hindering fracture healing, titanium alloy has replaced stainless steel in internal fixation (Hashemi et al., 2021; Claes, 2021; Benli et al., 2008; Röderer et al., 2014). Despite this, titanium’s modulus remains significantly higher than bone. Researchers are exploring composite materials with better biomechanical properties for bone plates (Roy et al., 2024). Meanwhile, optimizing the plate structure can enhance its biological fixation properties without changing the materials. Currently, an increasing number of scholars recognize that overly rigid fixation can induce asymmetric osteogenesis, thereby impeding rapid fracture healing and potentially increasing the risk of refracture. Consequently, their focus has shifted toward developing internal fixation devices that distribute strain uniformly across the proximal and distal cortical surfaces of the fracture site, in order to facilitate faster healing (Huxman et al., 2025a; Bullock et al., 2025; Huxman et al., 2025b; Huxman et al., 2026).

This study assessed the MVFP plate’s biomechanical performance under extreme conditions without bony support at the fracture ends. With a 1x body weight axial load, the average relative displacements were 0.33 ± 0.06 mm for MVFP and 0.28 ± 0.02 mm for LP. The optimal shim thickness for MVFP ranged from 0.2–0.67 mm after accounting for elastic deformation. Under a 700 N axial load, LP exhibited 0.28 mm of micromotion due to elastic deformation, which is less controllable and may lead to fatigue fractures. LP also had non-uniform micromotion, impairing callus formation. In contrast, MVFP ensured uniform, beneficial micromotion and had lower stiffness, reducing stress shielding. Simulated functional exercises showed no failures in the MVFP group, with plastic deformation similar to that of the LP group, indicating comparable fatigue performance 3 months post-implantation.

Simulations of magnesium shim degradation revealed that a 0.5 mm MVFP shim provided greater stiffness, leading to more stable early fixation. Further research is needed to explore if using stiffer materials like stainless steel could enhance MVFP stiffness and reduce uncontrolled deformation. Michael Plecko et al. found variable fixation plates better for healing sheep tibial fractures than traditional locked plates, with bilateral fixation being more effective than unilateral fixation due to increased axial micromotion (Plecko et al., 2020). Qiugen Wang et al. observed that micromotional plates (0.3 mm and 0.6 mm) were less strong than locked plates but more effective for healing, with the 0.6 mm plate outperforming the 0.3 mm version (Han et al., 2020). Thus, a 0.5 mm micromotion gap in MVFP ensures effective axial micromotion between fracture ends, making it a suitable choice for MVFP.

Discrepancies between *in vitro* mechanical simulations and FEA, largely due to variations in femoral models, do not undermine the reliability of the results or conclusions (Welch-Phillips et al., 2020; Lagravère, 2021; Lewis et al., 2021). It is crucial to recognize that FEA and *in vitro* simulations serve different purposes: FEA analyzes stress distribution and identifies potential design flaws, while *in vitro* experiments assess the actual performance of external fixatives. Both methods complement each other through mutual verification.

## 5 Conclusion

In conclusion, this study demonstrates that the MVFP provides a biomechanically viable alternative to conventional locking plates. Finite element analysis revealed that MVFP with a 0.5 mm shim offers comparable deformation and stress profiles under axial, bending, and torsional loading, while *in vitro* tests confirmed its slightly reduced but sufficient stiffness (81.5% axial, 68.5% bending, 63.9% torsional relative to LP). Importantly, MVFP maintained excellent fatigue resistance over 100,000 cycles and facilitated more uniform interfragmentary micromotion, which is beneficial for fracture healing. The degradable shim enables a transition from rigid to flexible fixation, reducing stress shielding and potentially enhancing callus formation. These outcomes support the further development and clinical testing of MVFP, particularly with a 0.5 mm micromotion gap, as a promising implant for stage-adaptive fracture fixation.

## Data Availability

The original contributions presented in the study are included in the article/supplementary material, further inquiries can be directed to the corresponding author.
